# Semaphorin 3A Inhibits Inflammation in Chondrocytes under Excessive Mechanical Stress

**DOI:** 10.1155/2018/5703651

**Published:** 2018-04-08

**Authors:** Chikako Sumi, Naoto Hirose, Makoto Yanoshita, Mami Takano, Sayuri Nishiyama, Yuki Okamoto, Yuki Asakawa, Kotaro Tanimoto

**Affiliations:** ^1^Department of Orthodontics and Craniofacial Developmental Biology, Hiroshima University Graduate School of Biomedical & Health Sciences, Hiroshima, Japan; ^2^Department of Orthodontics, Division of Oral Health and Development, Hiroshima University Hospital, Hiroshima, Japan

## Abstract

**Background:**

Excessive mechanical stress causes inflammation and destruction of cartilage and is considered one of the cause of osteoarthritis (OA). Expression of semaphorin 3A (Sema3A), which is an axon guidance molecule, has been confirmed in chondrocytes. However, there are few reports about Sema3A in chondrocytes, and the effects of Sema3A on inflammation in the cartilage are poorly understood. The aim of this study was to examine the role of Sema3A in inflammation caused by high magnitude cyclic tensile strain (CTS).

**Methods:**

Expression of Sema3A and its receptors neuropilin-1 (NRP-1) and plexin-A1 (PLXA1) in ATDC5 cells was examined by Western blot analysis. ATDC5 cells were subjected to CTS of 0.5 Hz, 10% elongation with added Sema3A for 3 h. Gene expression of IL-1*β*, TNF-*ɑ*, COX-2, MMP-3, and MMP-13 was examined by qPCR analysis. Furthermore, the phosphorylation of AKT, ERK, and NF-*κ*B was detected by Western blot analysis.

**Results:**

Added Sema3A inhibited the gene expression of inflammatory cytokines upregulated by CTS in a dose-dependent manner. Addition of Sema3A suppressed the activation of AKT, ERK, and NF-*κ*B in a dose-dependent manner.

**Conclusions:**

Sema3A reduces the gene expression of inflammatory cytokines by downregulating the activation of AKT, ERK, and NF-*κ*B pathways in ATDC5 cells under CTS.

## 1. Introduction

Mechanical stress is an important regulator of chondrocyte metabolism [1]. Mechanical loading within the physiological range is necessary for maintaining cell homeostasis. Cartilage tissue can remodel its extracellular matrix in response to alterations in various functional demand [2, 3]. On the other hand, an excessive mechanical loading over the physiological range causes abnormal inflammation through the reduction of matrix components and the synthetic imbalance between matrix metalloproteinases (MMPs) and tissue inhibitor of matrix metalloproteinases (TIMPs) finally leads to cell destruction [4, 5].

Semaphorins are a large family of secreted and membrane-bound glycoproteins. They were initially identified as regulatory molecules that define the direction of axonal growth. To date, over 20 members, classified into eight subclasses in terms of structural differences, have been identified. Class 3 semaphorins are vertebrate-secreted proteins and include seven members, semaphorin3A (Sema3A) to Semaphorin3G (Sema3G) [6]. Sema3A binds neuropilin-1 (NRP-1) and plexin-A1 (PLXA1) and also has a high affinity for the NRP-1/PLXA1 complex, interactions that activate signal transduction. It is known that the inhibition of Sema3A regulates the direction of axonal growth [7]. Sema3A and its receptors play pivotal roles in not only the nervous system but also the immune, cardiovascular and respiratory systems, organogenesis, tumorigenesis, and bone metabolism [8–12]. In addition, it was recently shown that NRP-1 and PLXA1 are present on the surface of chondrocytes; however, little is known about their roles [13].

The purpose of this research is to elucidate the function of Sema3A in inflammation and cartilage destruction using chondrocytes under high magnitude cyclic tensile strain (CTS).

## 2. Materials and Methods

### 2.1. Cell Line and Culture Conditions

A clonal chondrogenic cell line ATDC5 (RIKEN Cell Bank, Tsukuba, Japan) derived from mouse embryonic carcinoma is an in vitro model that is widely used to study chondrocyte growth and differentiation in vitro [14, 15]. Cells were plated in 6-well BioFlex culture plates (Flexcell International, Hillsborough, NC, USA) precoated with type *α* collagen at a density of 6.0 × 10^4^ cells/well. The culture was maintained in 2 ml of DMEM/Ham's F12 hybrid medium (Sigma, St. Louis, MO, USA) containing 10% fetal bovine serum (FBS; BioWhittaker, Verviers, Belgium), 10 mg/ml human transferrin (Sigma), and 3 × 10^−8^ M sodium selenite (Sigma). The medium was supplemented with ascorbate 2-phosphate (37.5 *μ*g/ml) (Sigma) and 10 *μ*g/ml bovine insulin (Sigma) to induce differentiation after the cells were grown for 7 days. The differentiation medium was changed every other day for 14 days. Cells were maintained at 37°C in a humidified atmosphere of 5% CO_2_ in air for the entire culture period.

### 2.2. Exposure to Mechanical Stress and Sema3A Application

High magnitude CTS was performed using the FX-2000 Flexcell system® (Flexcell International, McKeesport, PA, USA). CTS was enforced at 10% elongation (0.5 Hz; 1 s on, 1 s off). ATDC5 cells not subjected to CTS were used as a control. After 1, 3, 6, 12, or 24 h stretching, the supernatant and the cells were collected and frozen at −80°C for further analyses. To examine the effect of Sema3A, cells treated with Sema3A (1, 10, or 100 ng/ml; recombinant mouse semaphorin 3A Fc chimera, R&D Systems Inc., Minneapolis, MN, USA) were subjected to CTS for 3 h. To examine the effect of Sema3A on AKT, ERK, and NF-*κ*B phosphorylation, cells treated with Sema3A (1, 10, or 100 ng/ml) were subjected to CTS for 1 h.

### 2.3. Quantitative Real-Time Polymerase Chain Reaction (qPCR)

Total RNA was isolated from cell cultures with TRIzol (Invitrogen Life Technologies Inc., Gaithersburg, MD, USA) reagent, following the manual's instructions. cDNA was generated with ReverTra Ace (Toyobo, Osaka, Japan). qPCR analysis was performed using SYBR Green PCR Master Mix (Toyobo) and a LightCycler System (Roche Diagnostics, Mannheim, Germany) to quantify target gene expression. The primer sets used are shown in Table 1. Relative gene expression levels were calculated based on the GAPDH housekeeping gene as an internal control. Normalized cycle threshold (Ct) values were compared relative to those of the controls. Data were calculated as relative expression by 2^−ΔCt^, where the cycle threshold is the beginning of logarithmic amplification and ΔCt is the difference of the target gene Ct subtracted from the reference gene Ct. At least three independent measurements were averaged.

### 2.4. Western Blot Analysis

Whole cell lysates were collected by adding Triton X-100 buffer (Roche Diagnostics). Protein concentration was determined by the Bradford protein assay (Bio-Rad Laboratories, Hercules, CA, USA). Equal amounts (30 *μ*g) of protein were run in each lane on a Tris-glycine gel using SDS-PAGE (Atto, Tokyo, Japan). After electrophoresis, the proteins were transferred to a polyvinylidene difluoride (PVDF) membrane using an i-Blot system (Invitrogen). The membrane was then blocked with 4% (*w*/*v*) Block Ace (Yukijirushi Nyugyo Inc., Tokyo, Japan) and probed with 1 : 1000 dilutions of Sema3A (Abcam, Cambridge, UK), NRP-1 (Abcam), PLXA1 (ECM Biosciences, Versailles, KY, USA), IL-1*β* (Cell Signaling Technology Inc., Beverly, MA, USA), MMP-13 (Abcam), phospho-AKT (CST), total-AKT (CST), phospho-ERK1/2 (CST), total-ERK1/2 (CST), phospho-NF-*κ*B (CST), and total-NF-*κ*B (CST) antibodies overnight at 4°C. Next, 1 : 20000 diluted horseradish peroxidase conjugated antimouse antibody or antirabbit antibody (GE Healthcare Life Sciences, Tokyo, Japan) was used as the secondary antibody. Protein expression was normalized to beta-actin. Signals were detected using an Odyssey® Blot Analyzer (M&S Techno Systems, Osaka, Japan).

## 3. Results

### 3.1. Expression of Sema3A, NRP-1, and PLXA1 in ATDC5 Cells

**Figure 1:**
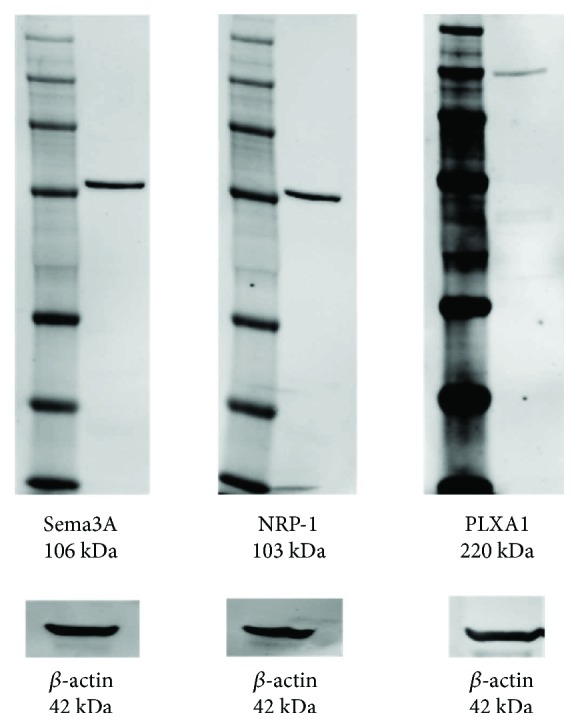
Expression of Sema3A, PLXA1, and NRP-1 in ATDC5 cells. Protein expression levels of Sema3A, PLXA1, and NRP-1 were determined in ATDC5 cells by Western blot analysis. *β*-actin was used as a loading control.

The expression in Sema3A, PLXA1, and NRP-1 proteins was confirmed in cultured ATDC5 cells. Western blot analysis of whole cell lysates using the Sema3A antibody showed a protein band of 106 kDa. NRP-1 and PLXA1 protein expression was also confirmed by immunoblotting, which showed a 103 kDa band for NRP-1 and 220 kDa band for PLXA1 (Figure 1).

### 3.2. Gene Expression Patterns of Sema3A and Its Receptors under Excessive CTS

**Figure 2:**
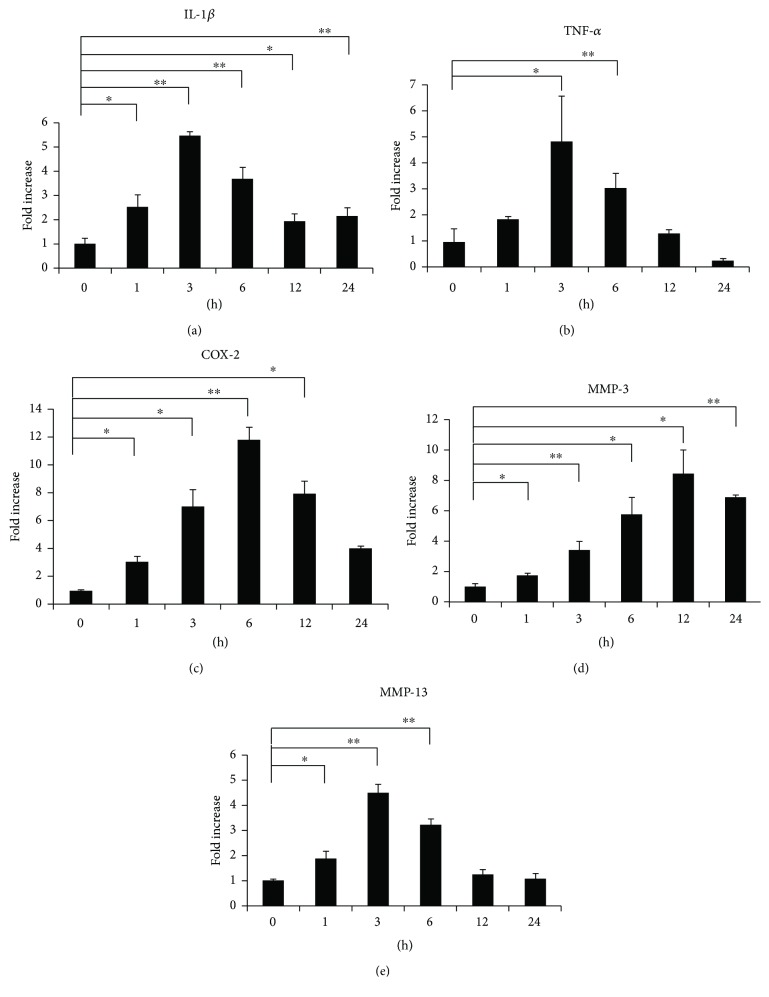
Change in gene expression patterns under excessive mechanical stress. ATDC5 cells were subjected to excessive CTS for 1, 3, 6, 12, or 24 h. Gene expression levels of IL-1*β*, TNF-*α*, COX-2, MMP-3, and MMP-13 at each time point were determined by qPCR analysis (a–e). Data are expressed as mean ± SD, *n* = 3. ^∗^ *P* < 0.05, ^∗∗^ *P* < 0.01 compared to controls at each time point.

**Figure 3:**
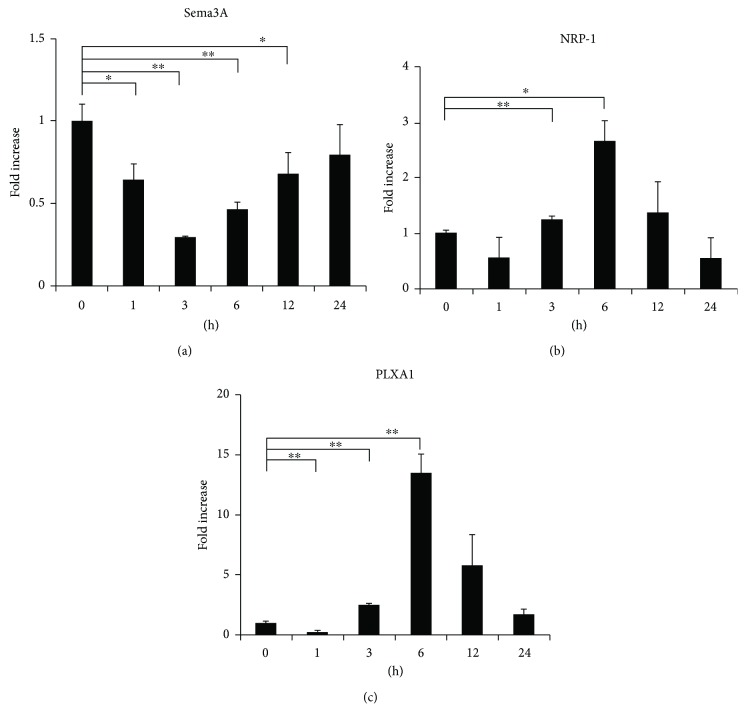
Change in gene expression patterns under excessive mechanical stress. ATDC5 cells were subjected to excessive CTS for 1, 3, 6, 12, or 24 h. Gene expression levels of Sema3A, NRP-1and PLXA1 at each time point were determined by qPCR analysis (a–c). Data are expressed as mean ± SD, *n* = 3. ^∗^ *P* < 0.05, ^∗∗^ *P* < 0.01 compared to controls at each time point.

To confirm changes in inflammatory mediators, Sema3A and its receptors, we examined mRNA expression levels using qPCR analysis. As shown in Figures 2(a)–2(e), mRNA expression of IL-1*β*, TNF-*α*, COX-2, MMP-3, and MMP-13 was significantly increased after loading. As shown in Figures 3(a)–3(c), the expression of Sema3A was markedly inhibited at 3 h after loading, and the expression of NRP-1 and PLXA1 was remarkably increased at 6 h after loading.

### 3.3. Effect of Sema3A on Expression of Inflammatory Mediators under Excessive CTS

**Figure 4:**
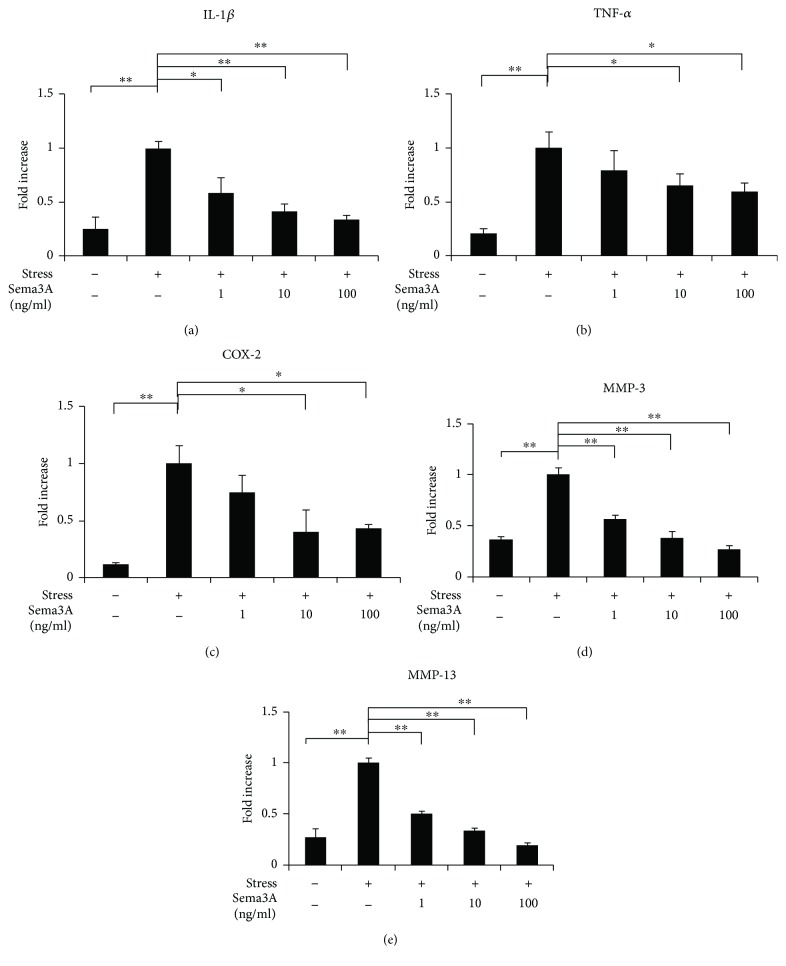
Effect of Sema3A on gene expression of inflammatory mediators under excessive mechanical stress. ATDC5 cells treated with various concentrations of Sema3A were subjected to excessive CTS for 3 h. Gene expression of IL-1*β*, TNF-*α*, COX-2, MMP-3, and MMP-13 was measured by qPCR (a–e). Data are expressed as mean ± SD, *n* = 3. ^∗^ *P* < 0.05, ^∗∗^ *P* < 0.01 compared to the group with CTS but without Sema3A.

**Figure 5:**
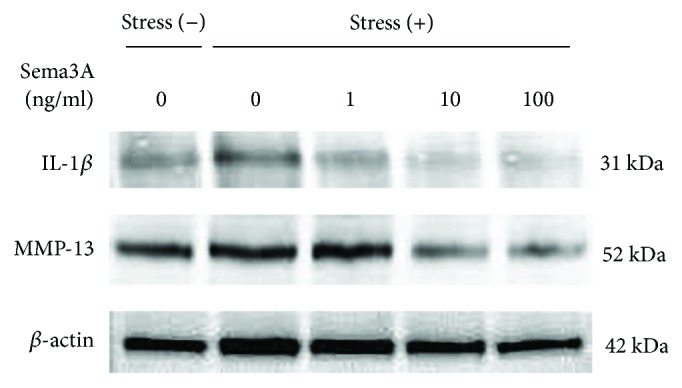
Effect of Sema3A on protein expression of IL-1*β* and MMP-13 under excessive mechanical stress. ATDC5 cells treated with various concentrations of Sema3A were subjected to excessive CTS for 24 h. Protein expression of IL-1*β* and MMP-13 was determined by Western blot analysis. *β*-actin was used as a loading control.

To investigate the effect of Sema3A on gene expression of inflammatory mediators under CTS for 3 h, we examined mRNA expression levels using qPCR analysis. As shown in Figures 4(a)–4(e), the addition of Sema3A significantly and dose-dependently inhibited gene expression of inflammatory mediators. We also examined protein expression of IL-1*β* and MMP-13 under CTS for 24 h using Western blot analysis. As shown in Figure 5, the addition of Sema3A significantly and dose-dependently inhibited expression of IL-1*β* and MMP-13.

### 3.4. Effect of Sema3A on Gene Expression of Rnd1 under Excessive CTS

**Figure 6:**
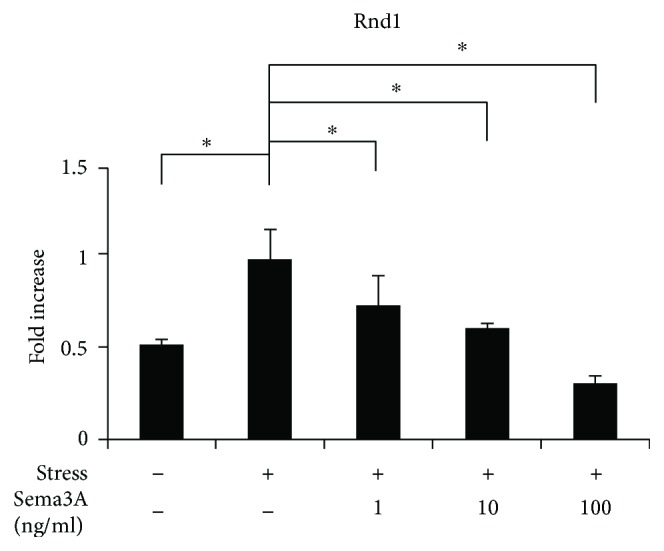
Effect of Sema3A on gene expression of Rnd1 under excessive mechanical stress. ATDC5 cells treated with various concentrations of Sema3A were subjected to excessive CTS for 3 h. Gene expression of Rnd1 was determined by qPCR analysis. Data are expressed as mean ± SD, *n* = 3. ^∗^ *P* < 0.05 compared to the group with CTS but without Sema3A.

To investigate the effect of Sema3A on gene expression of Rnd1 under CTS for 3 h, we examined mRNA expression levels using qPCR analysis. As shown in Figure 6, the addition of Sema3A significantly inhibited the expression of Rnd1.

### 3.5. Effect of Sema3A on Activation of AKT, ERK, and NF-*κ*B Phosphorylation

**Figure 7:**
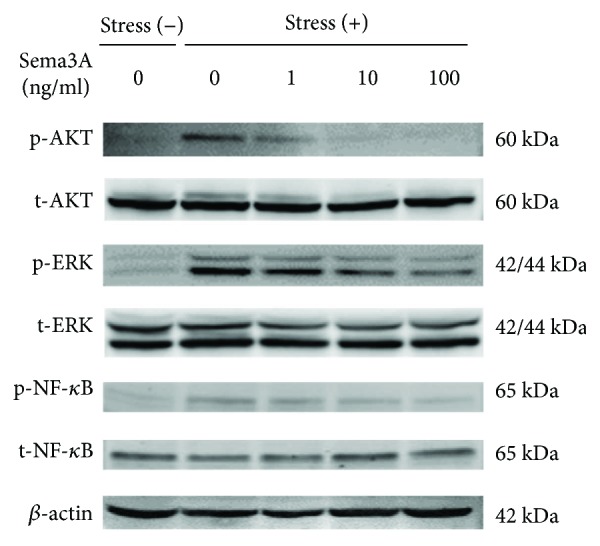
Effect of Sema3A on activation of AKT, ERK, and NF-*κ*B phosphorylation. ATDC5 cells treated with various concentrations of Sema3A (1, 10, or 100 ng/ml) were subjected to CTS for 1 h. Whole cell lysates were subjected to Western blot analysis.

To verify whether AKT, ERK, and NF-*κ*B are activated by excessive loading, we measured the expression of p-AKT, p-ERK, and p-NF-*κ*B in ATDC5 cells under CTS for 1 h. As shown in Figure 7, the phosphorylation of AKT, ERK, and NF-*κ*B was upregulated by CTS according to Western blot analysis. However, the phosphorylation of AKT, ERK, and NF-*κ*B was slightly blocked by 1.0 ng/ml of Sema3A and further inhibited by higher concentrations.

## 4. Discussion

This is the first study to show the role of Sema3A in inflammation under excessive periodic tensile loading in chondrocytes. Sema3A is expressed in various cell and tissue types, including the central nervous system, endothelial cells [16], intervertebral discs [17], heart [18], kidney [19], and bone [20]. The recent literature suggests that Sema3A is expressed by prehypertrophic and hypertrophic chondrocytes during endochondral ossification which precedes neurovascular invasion [21]. In this study, expression of Sema3A and its receptors was also confirmed in ATDC5 cells (Figure 1). The following have reported on the role of Sema3A in inflammation. Sema3A reduces cardiac inflammation by regulating cardiac monocyte/macrophage function in response to ischemia-induced myocardial injury [22]. Topically applied Sema3A ointment inhibits scratching behavior and improves skin inflammation in NC/Nga mice with atopic dermatitis [23]. Sema3A generated a significant increase in NO production through ERK and NF-*κ*B activation in the central nervous system-derived BV-2 microglial cell line, thereby regulating neuroinflammation [24]. However, the role of Sema3A with respect to inflammation in chondrocytes has not yet been clarified.

Physiological mechanical stress is required to maintain homeostasis of the extracellular matrix (ECM), but excessive mechanical stress causes the destruction of extracellular components [25]. Several studies have shown that excessive CTS induces inflammatory cytokines in chondrocytes [4, 26]. It has also been reported that IL-1 and TNF-*α*, which are important inflammatory cytokines involved in the induction of cartilage catabolism in OA, induce COX-2, MMP-3, and MMP-13 in chondrocytes [27–29].

In the present study, we found that expression of inflammation-related factors was increased significantly after loading (Figures 2(a)–2(e)). Moreover, mRNA expression of Sema3A was markedly decreased at 3 h after loading (Figure 3(a)). It has been reported that Sema3A is upregulated in human OA articular cartilage and that the Sema3A expression is closely correlated with chondrocyte cloning [13]. We assume that the upregulation of Sema3A is due to the compensatory responses from our results. Based on this result, we considered that Sema3A may be related to the onset of inflammation under excessive CTS. Meanwhile, the expression of NRP-1 and PLXA1 was increased with a slight delay (Figures 3(b) and 3(c)). Therefore, increased expression of the receptors is thought to compensate for decreased Sema3A expression. Furthermore, interestingly, the application of Sema3A strongly inhibited the production of inflammatory cytokines in a dose-dependent manner (Figures 4 and 5). The results showed that Sema3A may act to suppress inflammation in chondrocytes.

Next, we investigated the signal transduction pathways involved in inducing inflammatory cytokine expression.

Sema3A does not bind directly to plexin and requires neuropilin as a coreceptor [30–32]. Sema3A activates the receptor complex as the signal-transducing subunit. Rnd1 binds to the cytoplasmic domain of PLXA1 and its interaction induces signal transduction by PLXA1. Rnd1 is one of the Rho family of small GTPases and has been reported to regulate cytoskeletal dynamics, cell proliferation, and survival [33, 34]. This signaling is induced by active Rnd1, even in the absence of Sema3A [35]. Rnd1 controls Rho in fibroblasts and Ras in neurons [36] and has also been reported to inactivate Ras and MAPK pathways in mammary gland cells [37]. In this study, expression of Rnd1 was increased almost simultaneously with inflammation-related factors, and expression of Rnd1 was decreased by the application of Sema3A (Figure 6).

We evaluated the phosphorylation levels of AKT, ERK, and NF-*κ*B using Western blot analysis to investigate the role of Sema3A in the AKT, ERK, and NF-*κ*B signaling pathways. It has been reported that upregulation of MMP-3 and MMP-13 by IL-1 activates MAPK pathways as well as NF-*κ*B transcription factors in articular chondrocytes [28]. In addition, IL-1*β* is responsible for the upregulation of inflammatory genes in human chondrocytes via activation of JNK or AKT and NF-*κ*B signaling pathways [38, 39]. Thus, the MAPK and NF-*κ*B signaling pathways are known to regulate the production of inflammation-related factors in different cell systems [40]. In this report, phosphorylated AKT, ERK, and NF-*κ*B were increased significantly under excessive CTS, and significant decreases were observed following the addition of Sema3A (Figure 7). These results suggest that Sema3A could inhibit the production of inflammation-related factors via AKT, ERK, and NF-*κ*B signaling pathways in ATDC5 cells under excessive CTS.

## 5. Conclusions

We showed that Sema3A reduces the gene expression of inflammatory cytokines by downregulating the activation of AKT, ERK, and NF-*κ*B pathways in ATDC5 cells under excessive CTS.

## Figures and Tables

**Table 1:** The primer sequences for qPCR analysis.

Gene		Sequence of primers (5′-3′)	Amplicon Size (bp)
Sema3A	Forward	CAGCCATGTACAACCCAGTG	154
	Reverse	ACGGTTCCAACATCTGTTCC	

NRP-1	Forward	GGAGCTACTGGGCTGTGAAG	187
	Reverse	ACCGTATGTCGGGAACTCTG	

PLXA1	Forward	GTGTGTGGATAGCCATCA	433
	Reverse	CCAGCCTCTCGAACACT	

IL-l*β*	Forward	TGTGCAAGTGTCTGAAGCAGCTATG	1001
	Reverse	ACACAGGCTCTCTTTGAACAGAATG	

TNF-*α*	Forward	CTCCCTCCAGAAAAGACACCATGA	982
	Reverse	CTGACCACTCTCCCTTTGCAGAAC	

COX-2	Forward	CAGTTTCTCTACAACAACTCCATC	161
	Reverse	TTCATCTCTCTGCTCTGGTC	

MMP-3	Forward	CTCAAGGGTGGATGCTGTC	234
	Reverse	TGCCATAGCACATGCTGAAC	

MMP-13	Forward	CC AAAAGAGGT GAAGAGACTGA	128
	Reverse	CGGGGATAATCTTTGTCCATA	

Rnd1	Forward	CATCAGCCGTCCAGAGACCA	111
	Reverse	CGAAGATCTGTCTTGCAGCCAATA	

GAPDH	Forward	ATCATCCCTGCATCCACT	156
	Reverse	GTCATCATACTTGGCAGGTTTC	

## Supplementary Materials

Supplementary Materials Figure S1: time course of marker gene expression during differentiation of ATDC5 cells. Cells were grown in 6-multiwell plates. Total RNA was isolated on the indicated day of culture and analyzed by real-time PCR analysis. Data are expressed as mean ± SD, *n* = 3. ^∗^ *P* < 0.05, ^∗∗^ *P* < 0.01, compared to controls at each time point. Figure S2: FX-2000 Flexcell system. Schematic diagram of the FX-2000 Flexcell system. Cultured cells stretched using the FX-2000 Flexcell system, which is a computer-controlled apparatus that creates a programmable biaxial strain across laminin-coated culture wells. A CTS resulting in 10% cell elongation was applied at a frequency of 0.5 Hz (alternative stimulation and relaxation for 1 s). The apparatus was kept at 37°C in a humidified incubator with 5% CO_2_.
