# Supplementary material for: Semaphorin 3A Inhibits Inflammation in Chondrocytes under Excessive Mechanical Stress
Source: Mediators Inflamm. 2018 Apr 8;2018:5703651. doi: 10.1155/2018/5703651 (PMC5911320; doi:10.1155/2018/5703651)
Supplement: Supplementary Materials — Figure S1: time course of marker gene expression during differentiation of ATDC5 cells. Cells were grown in 6-multiwell plates. Total RNA was isolated on the indicated day of culture and analyzed by real-time PCR analysis. Data are expressed as mean ± SD, n = 3. ∗ P < 0.05, ∗∗ P < 0.01, compared to controls at each time point. Figure S2: FX-2000 Flexcell system. Schematic diagram of the FX-2000 Flexcell system. Cultured cells stretched using the FX-2000 Flexcell system, which is a computer-controlled apparatus that creates a programmable biaxial strain across laminin-coated culture wells. A CTS resulting in 10% cell elongation was applied at a frequency of 0.5 Hz (alternative stimulation and relaxation for 1 s). The apparatus was kept at 37°C in a humidified incubator with 5% CO2. [file 5703651.f1.docx]

**
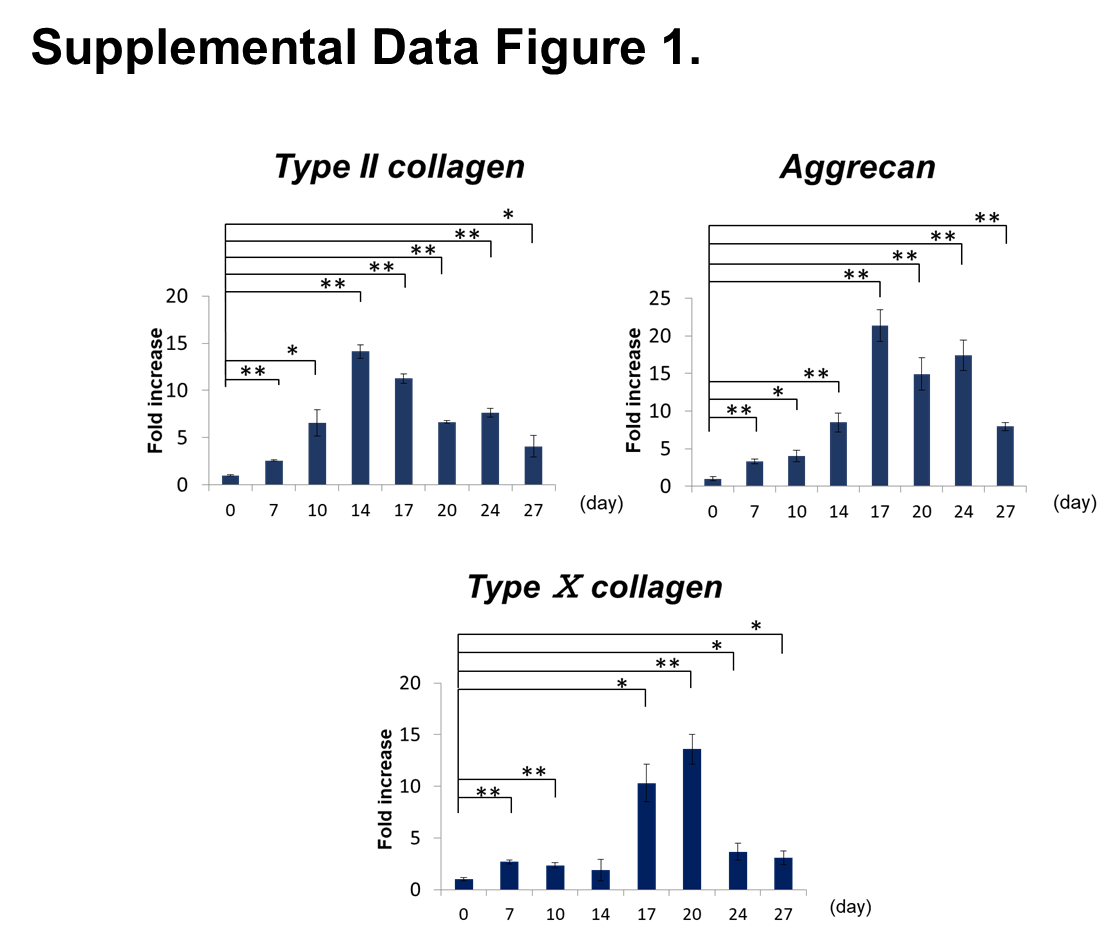
**

**Figure S1. Time course of marker gene expression during differentiation of ATDC5 cells.**

Cells were grown in 6-multiwell plates. Total RNA was isolated on the indicated day of culture and analyzed by real-time PCR analysis. Date are expressed as mean ± SD, n=3. *: P < 0.05, **: P < 0.01, compared to controls at each time point.


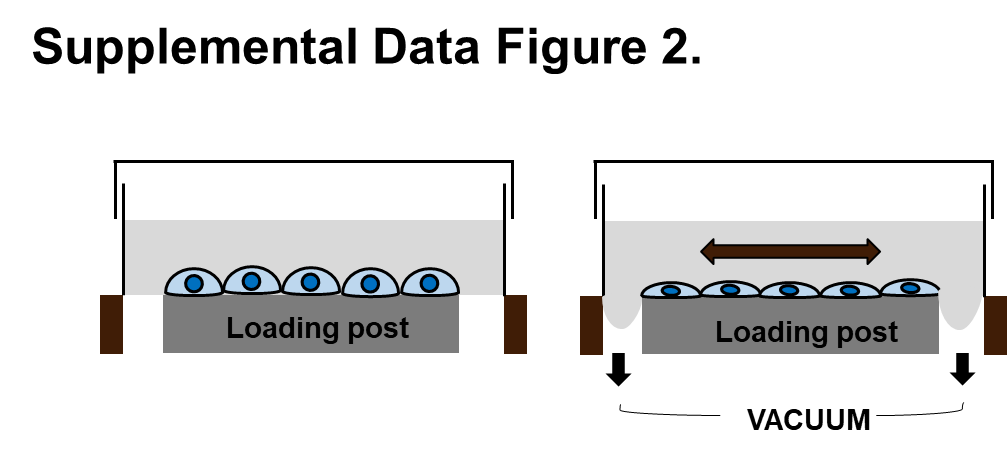


**Figure S2. FX-2000 Flexcell system^®^**

Schematic diagram of the FX-2000 Flexcell system^®^. Cultured cells were stretched using the FX-2000 Flexcell system^®^, which is a computer-controlled apparatus that creates a programmable biaxial strain across laminin-coated culture wells. A CTS resulting in 10% cell elongation was applied at a frequency of 0.5 Hz (alternative stimulation and relaxation for 1 s). The apparatus was kept at 37°C in a humidified incubator with 5% CO_2_.
